# Antioxidant potential of sulfenimides in waste walnut oil biodiesel: kinetic stability, phosphomolybdenum activity, and reducing power capacity

**DOI:** 10.55730/1300-0527.3777

**Published:** 2026-01-04

**Authors:** Nalan TÜRKÖZ KARAKULLUKÇU, Hasan YAKAN, Volkan Murat YILMAZ, Halit MUĞLU, Semiha YENİGÜN, Halil KÜTÜK

**Affiliations:** 1 Karadeniz Advanced Technology Research and Application Center, Ondokuz Mayıs University, Samsun, Turkiye; 2 Department of Chemistry Education, Faculty of Education, Ondokuz Mayıs University, Samsun, Turkiye; 3 Central Research Laboratory, Bartin University, Bartın, Turkiye; 4 Department of Chemistry, Faculty of Science, Kastamonu University, Kastamonu, Turkiye; 5 Department of Chemistry, Faculty of Science, Ondokuz Mayıs University, Samsun, Turkiye

**Keywords:** Sulfenimides, kinetic study, antioxidant, biodiesel, inhibitory effect

## Abstract

This study investigated the influence of synthetic sulfenimides on biodiesel–diesel fuel blends. Three previously synthesized sulfenimide derivatives were structurally characterized using spectroscopic techniques. We evaluated their antioxidant properties through phosphomolybdenum activity and reducing power capacity assays. The tested fuel samples were designated as D100, B10D90, B10D90_AA (ascorbic acid), B10D90_**3**, B10D90_**2**, and B10D90_**1**. The effect of adding 2000 ppm of the additive was measured using an Oxifast instrument following the ASTM D7545 standard, and the results were compared to those of the chemical antioxidant ascorbic acid. Inhibition time values were derived from the oxidation stability results. The starting temperatures for crystallization (Tc) of the mixtures were found using differential scanning calorimetry (DSC), and the measured temperatures were −7.24, −7.64, −7.89, −8.06, −8.39, and −8.52 °C. FT-IR spectra exhibited characteristic absorption bands associated with antioxidant functional groups, which means the sulfenimide compounds were successfully added. The addition of sulfenimides improved the oxidative stability of biodiesel blends. Furthermore, we conducted thermogravimetric analysis (TGA) at multiple heating rates to investigate the thermal decomposition kinetics of the sulfenimides. The activation energies for compounds **1**, **2**, and **3** were calculated using the Kissinger–Akahira–Sunose (KAS) method and were found to be 125.28, 111.34, and 88.11 kJ mol^−1^, respectively.

## 1 Introduction

Biodiesel has gained popularity as an alternative fuel source in recent years because it can lessen greenhouse gas emissions and reliance on fossil fuels [1–3]. However, one of the challenges associated with biodiesel is its tendency to crystallize at low temperatures [4–6], leading to filter clogging and engine performance issues. To address this issue, researchers have been exploring various additives that can improve the cold-flow properties of biodiesel [7–9].

Sulfenimides are widely used in various industrial applications, including insecticides, fungicides, and rubber vulcanization processes [10,11]. Phthalimide ring systems are highly prevalent in a range of industrial, agricultural, and medical applications [12,13]. These compounds have found extensive use due to their unique properties and versatile nature.

**Figure 1:**
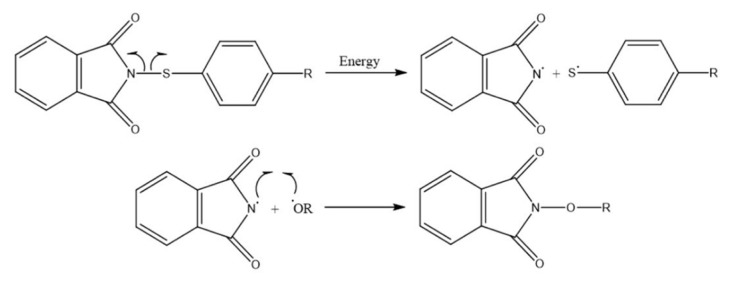
Synthetic method for sulfenimide radical mechanism [11,21].

In the context of this study, the phthalimide ring system plays a significant role in pharmacokinetic properties [14] and antifungal agents [15]. These agents, commonly known as synthetic commercial fungicides, are readily available on the market [16]. They have utilized molecules with versatile applications as vulcanization accelerators [17] and sulfur-transfer reagents [18]. In this study, the focus was on sulfenimide derivatives, which have shown promise in enhancing the performance of biodiesel-diesel blends. The main objective of the research was to investigate the effects of incorporating sulfenimide derivatives into biodiesel-diesel blends on their crystallization temperatures. The researchers hypothesized that adding these derivatives would lower the blends’ crystallization temperature, thereby improving their cold flow properties [19]. The study focused on sulfenimide derivatives and their impact on the crystallization point of biodiesel–diesel blends, revealing a previously uninvestigated function of these compounds as cold-flow improvers. Sulfenimides, a class of compounds known for their diverse applications in various industries, have long been a subject of interest for scientists [11,20,21]. Conducted a study to examine the impact of sulfenimide derivatives on the crystallization point of biodiesel utilizing sophisticated methodologies. Sulfenimide enhances the oxidation stability of biodiesel by disrupting the radical chain reaction and limiting the formation of new radicals [21–23]. This addition is intended to inhibit the formation of oxidative processes involving sulfenimide radicals. The sulfenimides begin this reaction, with the phthalimide radical serving as a very efficient leaving group. Figure 1 illustrates the formation of sulfenimide radicals under favorable conditions and their subsequent merging with RO. radicals [21]. Sulfenimide derivatives were added, and a concentration of 2000 parts per million (ppm) was added to the mixtures. Differential scanning calorimetry (DSC) and assays were used to evaluate the antioxidant activity of sulfenimide derivatives.

Figure 1 demonstrates that the homolytic breakage of the S–N link in sulfenimides produces phthalimide-centered and thiyl radicals in oxidative circumstances. These highly reactive sulfur centered species effectively capture peroxyl (ROO^•^) and alkoxyl (RO^•^) radicals, therefore inhibiting the chain propagation processes that lead to lipid peroxidation and total fuel oxidation [11,21]. This inherent radical-scavenging mechanism explains the significant antioxidant activity reported in sulfenimide enriched biodiesel-diesel mixtures.

The study involved comparing the experiment’s findings with the results achieved from using ascorbic acid. Apart from its position as a vital water-soluble antioxidant and reducing agent, ascorbic acid can potentially improve vitamin E’s effectiveness [24]. The involvement of ascorbic acid in the regeneration or restoration of vitamin is thought to contribute to this effect. Vitamin C functions as an electron donor, making it a reducing agent. The physiological and biochemical effects of vitamin C are solely attributed to its role as an electron donor [25]. Ascorbic acid contributes two electrons by breaking the double bond between the second and third carbons of the 6-carbon molecule [24]. Vitamin C is classified as an antioxidant due to its ability to donate electrons inhibiting other substances’ oxidation [26]. Nevertheless, due to the inherent characteristics of this reaction, vitamin C undergoes oxidation during the process. Table 1 displays the antioxidant properties and structural formula of ascorbic acid. Ascorbate dominates biological pH levels [27,28]. It functions as a gentle reducing agent and antioxidant. The substance undergoes oxidation by losing one electron, resulting in the formation of a radical cation. Subsequently, it loses a second electron, leading to the formation of dehydroascorbic acid. It usually responds to oxidants that belong to the reactive oxygen species, such as the hydroxyl radical [29,30]. Ascorbic acid possesses a unique property of being able to transport a solitary electron, thanks to the resonance-stabilized characteristic of its radical ion, known as semidehydroascorbate [31,32]. The overall reaction is given in Equation 1.


(1) $$RO^{.}+C_{6}H_{7}{O_{6}}^{-}\to RO^{-}+C_{6}H_{7}{O_{6}}^{.}\to ROH+C_{6}H_{6}O_{6}$$

DSC analyses were performed to determine the crystallization onset temperatures of all samples, and the results were plotted as heat flow versus temperature curves DSC is a valuable technique for monitoring crystallization behavior, which is essential for characterizing the cold-flow properties of biodiesel samples composed of long-chain fatty acid methyl esters [6,19]. DSC-based evaluation of the crystallization phenomenon yields valuable data on biodiesel samples with varying carbon chain lengths and degrees of unsaturation [33,34]. The crystallization behavior of esters depends on the length of the fatty acid chains and intermolecular interactions. The crystallization behavior differs between saturated and unsaturated methyl esters [35]. This is due to the molecular structure of the esters, particularly the chain length and the position of double bonds. The crystallization temperature tends to decrease with increased branching in the carbon chains of esters [4,34,35,36]. Furthermore, unsaturation has a significant effect on crystallization temperature. The spatial arrangement of unsaturated molecules disrupts molecular packing, resulting in lower crystallization temperature [36].

This is the first comprehensive study to employ structurally defined sulfenimide derivatives as dual-function additives in biodiesel–diesel combinations, to the best of our knowledge. This work introduces a new class of sulfur-nitrogen (S–N) bonded compounds that can both enhance oxidative stability and improve cold-flow performance, unlike prior papers that mostly focused on plant-derived or Schiff base antioxidants. The combined use of DSC, TGA–KAS kinetic modeling, FT-IR, and in vitro antioxidant assays provides a thorough evaluation of their physicochemical characteristics. This integrative approach clarifies the radical trapping mechanism of sulfenimides and verifies their thermal stability, offering a practical and synthetically viable pathway for the development of next-generation biodiesel additives.

## 2 Materials and methods

### 2.1. Materials

In the study, various compounds were utilized in their as-received form without the need for additional purification. The starting reagents and solvents for these compounds were sourced from well-known suppliers such as Merck (Germany), Sigma (USA), and Aldrich (USA) Chemical Company. Diesel fuel (D100) supply was provided from OPET company in Samsun, Türkiye. On the other hand, Aves Energy Oil and Food Industry (Mersin, Türkiye) received a shipment of biodiesel, known as B100, produced entirely from waste walnut oil. This allocation of different fuel types marks an important step in our ongoing efforts to explore alternative energy sources and reduce our reliance on traditional fossil fuels.

### 2.2. Methods

#### 2.2.1. Sulfenimide synthesis

**Figure 2:**
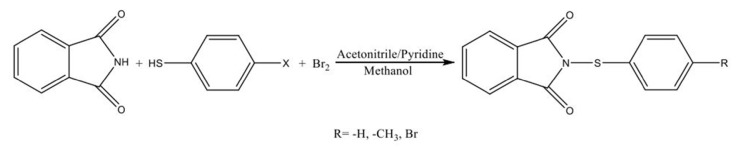
Synthetic route method for sulfenimide derivatives.

Thiophenol (5.25 mmol) or a derivative and phthalimide (5.25 mmol) are heated in a combination of acetonitrile and pyridine to dissolve the compounds. The reaction mixture was allowed to cool to ambient temperature. The bromide is dissolved in acetonitrile and the bromide solution is then added dropwise at room temperature and stirred for 30 min. Following a 2-h period of mixing at room temperature, introduce 40 mL of methanol and allow it to react for 30 min. The resultant solid product is stored in a water-ice bath for 30 min and subsequently transformed into methanol/ethanol crystals. IR, ^1^H NMR, ^13^C NMR, and DSC techniques were utilized to confirm the chemical structures of products. The synthetic route method and physical data of sulfenimide derivatives are given in Figure 2 and Table 2, respectively [20,21,37].

*N*-(*p*-Methylphenylthio)phthalimide (**1**): FT-IR (KBr, cm^−1^): υ(Ar C–H) 3103–3086, υ(C=O) 1746–1720, υ(C–N) 1173, υ(S–N) 1084, υ(C–S) 721, υ(–CH) 2936–2852; ^1^H NMR(CDCl_3_): δ/ppm = 2.36 (CH_3_,3H, s), 7.13–7.17 (2H, d), 7.56–7.60 (2H, d), 7.78–7.83 (2H, dd *J*=7.86 Hz), 7.84–7.90 (2H, dd *J*=8.24 Hz); ^13^C NMR(CDCl_3_): δ/ppm = 21.8, 123.9,129.4, 131.3, 131.5, 132.8, 134.5, 142.7, and 167.7 [39].

*N*-(Phenylthio)phthalimide (**2**): FT-IR (KBr, cm^−1^): υ(Ar C–H) 3065–3035, υ(C=O) 1736–1709, υ(C–N) 1153, υ(S–N) 1067, υ(C–S) 739; ^1^H NMR(CDCl_3_): δ/ppm =7.23–7.90 (arom-H, 9H, m); ^13^CNMR(CDCl_3_): δ/ppm =125.0, 128.3, 130.9,131.3, 131.4, 134.6, 134.7, and 167.7 [39].

*N*-(Bromophenylthio)phthalimide (**3**): FT-IR (KBr, cm^−1^): υ(Ar C–H) 3168–3059, υ(C=O) 1775–1730, υ(C–N) 1138, υ(S–N) 977, υ(C–S) 745, υ(Ar–Br) 644; ^1^H NMR (CDCl_3_): δ/ppm = 7.27–7.37 (d, 2H), 7.37–7.48 (d, 2H), 7.77–7.79 (t, 4H), 7.91–7.93 (t, 4H); ^13^C NMR (CDCl_3_): δ/ppm = 123.5, 131.9, 132.5, 132.8, 134.0, 136.3, 136.8, and 167.5 [39].

#### 2.2.2. Preparations of biodiesel-diesel blends

The biodiesel-to-diesel ratio in mixes of diesel and biodiesel typically ranges from 10% to 90%. The mixture being combined is indicated by the chemical formula B10D90. The mixture was carefully blended at a concentration of 2000 parts per million (ppm). The concentration was chosen to attain maximum efficacy and to optimize the potential benefits of the combined mixture.

#### 2.2.3. Differential scanning calorimetry (DSC)

**Figure 3:**
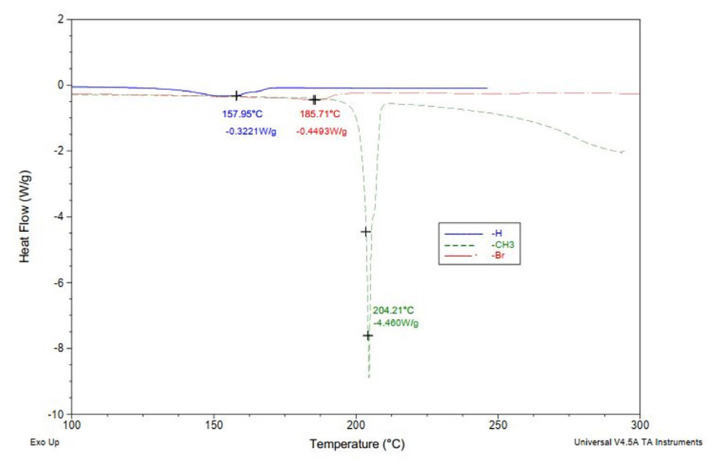
DSC curves of *N*-(phenylthio)phthalimide (**2**), *N*-(*p*-methylphenylthio)phthalimide (**1**), and *N*-(*p*-bromophenylthio)phthalimide (**3**).

Differential Scanning Calorimetry (DSC) was used to investigate the melting behavior of organic blends. This analytical technique provides detailed insights into the thermal behavior of materials, particularly their phase transitions such as melting and crystallization. During the controlled heating process, DSC measures the heat flow associated with endothermic or exothermic events, enabling precise determination of transition points [33,36,40,41]. Sulfenimide samples (~10 mg each) were weighed into aluminum pans and subjected to a heating protocol from 25 °C to 300 °C at a rate of 10 °C/min under a nitrogen atmosphere. This method enabled the identification of specific melting temperatures for each compound, ensuring reliable thermal analysis. The corresponding DSC thermograms are presented in Figure 3.

DSC techniques may be employed to characterize, quantify, and infer [42]. The main aim of this study was to examine the temperatures at which the process of crystallization begins for the materials. The experiment was conducted using a TA Q-2000 calorimeter (California, USA), which was fitted with an RCS90 and a cooling system. The thermal behavior of sulfenimides was analyzed using the DSC technique. The sample was cooled at a rate of 10 °C/min in an aluminum pan from an initial temperature of 25 °C to a final temperature of −90 °C, with approximately 10 mg of the sample being used. The reference was made with empty metal pans. High-purity nitrogen was delivered at a flow rate of 50 mL/min as the purge gas.

#### 2.2.4. Fourier transform infrared spectroscopy (FT-IR)

Recent research utilized Fourier transform infrared spectroscopy (FT-IR), a sophisticated analytical method, to thoroughly analyze the chemical functional groups [43]. The research was conducted using the Perkin Elmer Spectrum-Two, an FT-IR device developed in the United States. Through the utilization of this cutting-edge technology. FTIR analysis revealed characteristic vibrational bands corresponding to functional groups, confirming the chemical integrity of the samples. To conduct a comprehensive investigation of the material, a surface scan was performed utilizing a spectral range of 650 to 4000 cm^−1^. The selection of this range was meticulously made to encompass a broad spectrum of wavelengths, enabling a thorough analysis of the material’s characteristics. By utilizing this specific spectrum of wavelengths, scientists were able to collect significant data and gain a profound understanding of the composition and properties of the item being studied. The investigation involved acquiring ATR-FTIR spectra using an isothermal approach at ambient temperature [44–46].

#### 2.2.5. Determination of inhibition time

The inhibition time data were obtained by converting the results obtained from the investigation of oxidation stability. The ASTM D7545 Standard Test Method for Oxidation Stability of Middle Distillate Fuels—Rapid Small Scale Oxidation Test (RSSOT) was employed to assess the oxidation stability. For this examination, a 5 mL sample was put in a hermetically sealed chamber at a temperature of 140 °C in an environment with only oxygen [41,47].

#### 2.2.6. Kinetics of sulfenimide derivatives

Two essential methods are used in the kinetics of solid decomposition: model fitting and the model-free method [48]. Fitting experimental data to the reaction model is not a good way to get kinetic information for nonisothermal studies because it is hard to tell the difference between k (T), which depends on temperature, and f (±), which is the reaction model. Many kinetic prediction methods propose the isoconversional (model-free) method to obtain reliable and consistent information. The model-free method enables the determination of activation energy as a function of conversion and temperature, disregarding the reaction model [49]. This study used the Kissinger-Akahira-Sunose (KAS), the most well-known model-free method, to calculate the activation energy required for the thermal decomposition of sulfenimide derivatives.

The kinetic study, based on thermogravimetric analysis results of sulfenimide derivatives, was performed using a Hitachi STA 7300 instrument (Tokyo, Japan) in a nitrogen atmosphere with a flow rate of 100 mL min^−1^ at different heating rates (5, 10, 15, 20 °C min^−1^), with approximately 5 mg of the compounds, and heating ranging from room temperature to 700 °C. Through Equation 2, the KAS method determines the activation energy of thermal decomposition.


(2) $$\text{ln }\left(\frac{\beta }{T^{2}}\right)=\text{ln }\left[\frac{E.A}{R.g(a)}\right]-\left(\frac{E}{R.T}\right)$$

(3) $$g(a)=\int_{0}^{a}f{\left(a\right)}^{-1}\;da$$

Where A represents the exponential factor with units of min^−1^, β is the heating rate (K min^−1^), R is the gas constant (8.314 J mol^−1^ K^−1^), T is the absolute temperature (K), E is the activation energy (J mol^−1^), and Equation 3 is the integral form of f(α). The activation energies for different α values might be determined by calculating the slope of the graph ln β/T^2^ versus 1000/T [50].


(4) $$k(T)=A.\text{exp }\left(\frac{-E}{RT}\right)$$

Where A denotes the pre-exponential factor with units of min^−1^, R is the universal gas constant valued at 8.314 J mol^−1^ K^−1^, and E signifies the activation energy expressed in J mol^−1^ (Equation 4). The kinetic triplet—comprising the values of E, A, and the reaction model function f(α) valuesis employed to predict the extent of conversion of a chemical reaction under specified temperature conditions [51].

#### 2.2.7. Phosphomolybdenum activity

The ammonium molybdenum method [52,53] determined the phosphomolybdenum activity of the samples dissolved in DMSO. 300 μL of sample or ascorbic acid at different concentrations was taken and mixed homogeneously with 2.7 mL of reagent solution (28 mM sodium phosphate + 4 mM ammonium molybdate + 0.6 M H_2_SO_4_). The mixture was stirred in a shaking water bath at 95 °C for one and a half h. After the samples and ascorbic acid solutions were transferred to a 96-well plate at room temperature, their absorbance was measured at 695 nm, and the values were calculated as A_0.5_ (μg/mL).

#### 2.2.8. Reducing power capacity

The reducing power capacities of samples dissolved in DMSO were investigated using Oyaizu’s (1986) method [54,55]. In a 96-well plate, 20 μL of sample or ascorbic acid at different concentrations was taken, and 50 μL of 0.2 M PBS (pH 6.6) and 50 μL of 1% K_3_FeCN_6_ solutions were added and incubated at 50 °C for 20 min. Fifty μL ddH_2_O, 50 μL 10% TCA, and 10 μL 0.1% FeCl_3_ solutions were added to the mixture and mixed thoroughly. A_0.5_ (μg/mL) values were determined using absorbance values measured at 700 nm.

#### 2.2.9. Statistical analysis

Analytical findings of antioxidant activity (in vitro) experiments obtained in triplicate were presented together with ± standard deviation values. The IBM-SPSS 20.0 software package was used to analyze all the data. A one-way ANOVA (Tukey HSD^a,b^) calculation was performed for multiple comparisons in line with the collected data. The statistical significance level of the values was considered statistically significant when compared to the activity analysis result group and was expressed as p < 0.05 values.

## 3 Results and discussion

### 3.1. Differential scanning calorimetry (DSC)

**Figure 4:**
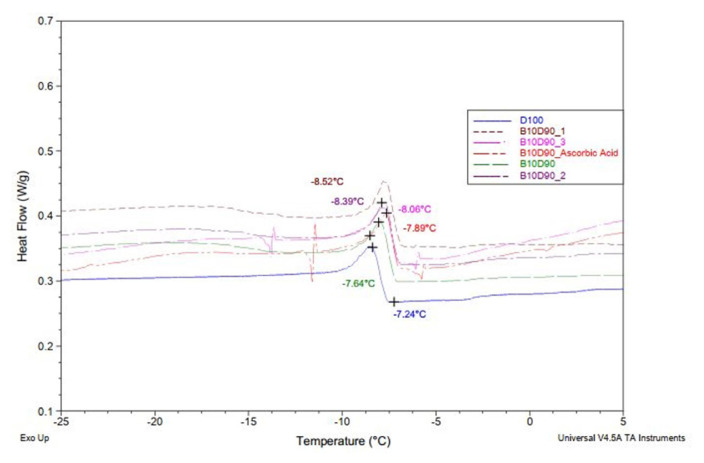
DSC thermograms of D100, B10D90, B10D90_AA, B10D90_**3**, B10D90_**2**, and B10D90_**1** under N_2_ atmosphere.

The aim of this study was to investigate the crystallization onset temperatures (Tc) of six biodiesel–diesel blends using differential scanning calorimetry (DSC). The tested samples included D100 (pure biodiesel), B10D90 (10% biodiesel + 90% diesel), B10D90_AA (ascorbic acid-supplemented blend), and three blends containing different sulfenimide derivatives: B10D90_**3**, B10D90_**2**, and B10D90_**1**. The DSC analysis revealed distinct crystallization behaviors among the samples. Measured Tc values were −7.24 °C (D100), −7.64 °C (B10D90), −7.89 °C (B10D90_AA), −8.06 °C (B10D90_**3**), −8.39 °C (B10D90_**2**), and −8.52 °C (B10D90_**1**), respectively. The progressive decrease in crystallization onset temperatures upon sulfenimide addition indicates improved cold-flow properties of the fuel blends. Figure 4 displays the heat flow curves obtained from the DSC measurements, while Table 3 summarizes the crystallization onset data. The findings underscore the significance of additive structure on the crystallization behavior of biodiesel blends and highlight the importance of precise thermal analysis in evaluating low-temperature fuel performance. The mean onset temperature for crystallization is (*χ*_mean_=|−7.96| °C). A standard deviation value of s = 0.46 °C has been determined, which is a common measure of the variability among experimental measurements according to Equation (5), as shown in Table 3.


(5) $$s^{2}=\frac{\Sigma_{i=1}^{N}{\left(xi-x_{mean}\right)}^{2}}{N-1}$$

**Figure 5:**
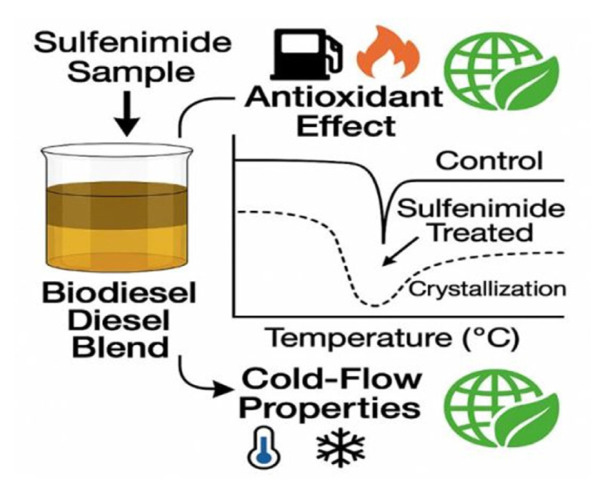
Addition of sulfenimide (2000 ppm) to biodiesel–diesel blend improved oxidative and cold-flow stability, as shown by the DSC thermogram shift.

As illustrated in Figure 5, the addition of the sulfenimide sample to the biodiesel–diesel blend altered the crystallization behavior, shifting the DSC peak to a lower temperature. This shift is attributed to the antioxidant and crystal-disruptive effects of the sulfenimide derivative, contributing to improved cold-flow properties.

### 3.2. Fourier transform infrared spectroscopy (FT-IR)

**Figure 6:**
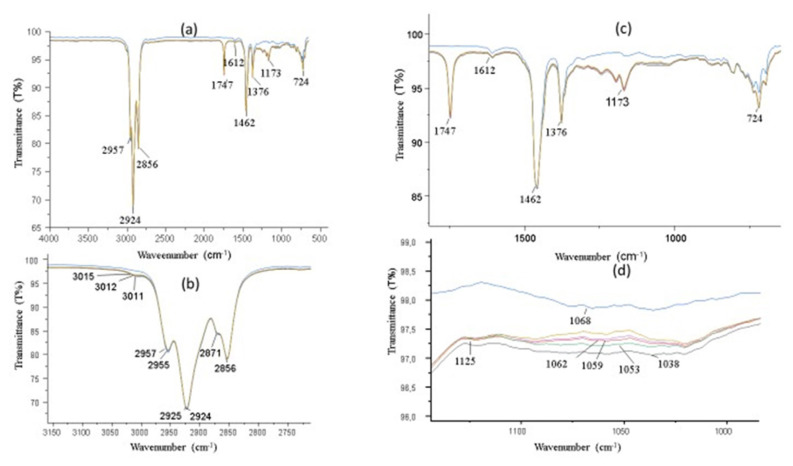
**(a)** FT-IR spectra of D100, B10D90, B10D90_AA, B10D90_**3**, B10D90_**2**, and B10D90_**1** at 4000–500 cm^−1^. **(b)** Antioxidant effects of D100, B10D90, B10D90_AA, B10D90_**3**, B10D90_**2**, and B10D90_**1** samples between wavenumbers 2750 and 3150 cm^−1^. **(c)** Antioxidant effects of D100, B10D90, B10D90_AA, B10D90_**3**, B10D90_**2**, and B10D90_**1** samples between wavenumbers 500 and 2000 cm^−1^. **(d)** Antioxidant effects of D100, B10D90, B10D90_AA, B10D90_**3**, B10D90_**2**, and B10D90_**1** samples between wavenumbers 1000 and 1150 cm^−1^.

We will discuss FT-IR values for biodiesel-diesel blends. Different data are required to determine the composition and attributes of various mixes. Figure 6 (a–d) will also display the FT-IR graphs, which provide a graphical depiction of the data. FT-IR is a method employed for the examination of the molecular constitution of a material. Measuring the absorption of infrared light can provide valuable information on the chemical bonds and functional groups of the sample. The FT-IR values are critical in understanding the blending of biodiesel and diesel mixes. The spectra confirmed the presence of characteristic functional groups, such as C=O, C–O, and O–H, validating the successful incorporation of sulfenimide and biodiesel constituents. The FT-IR spectra of the sulfenimide-modified biodiesel blends demonstrate constant molecular interactions between the additives and the biodiesel matrix. While the general spectrum patterns resemble those of pure biodiesel due to the prevalence of aliphatic and ester absorptions, a further examination reveals notable alterations in band shape and relative intensity within the typical stretching areas. These alterations denote particular interactions between the polar functional sites of sulfenimides and the ester carbonyl and ether links of biodiesel. Furthermore, the emergence of supplementary low-frequency shoulders linked to S–N and S–C vibrations reinforces the chemical integration of sulfenimides rather than their physical coexistence. The FT-IR results collectively affirm that the additives are uniformly incorporated into the biodiesel–diesel blends, resulting in a stable and chemically compatible system.

FT-IR (ATR, cm^−1^): B10D90_**1**: υ (arom. C−H) 2957, υ (alif. C−H) 2871, υ(C=O) 1747, υ(N−H) 1462, υ(C−N) 1173, υ(C−O) 1068; B10D90_**2**: υ (arom. C−H) 2955, υ (alif. C−H) 2871, υ(C=O) 1747, υ(N−H) 1346, υ(C−N) 1125, υ(C−O) 1049; B10D90_**3**: υ (arom. C−H) 2955, υ (alif. C−H) 2871, υ(C=O) 1747, υ(N−H) 1746, υ(C−N) 1125, υ(C−O) 1049; B10D90_AA: υ(O−H) 3015, υ (arom. C−H) 2925, υ (alif. C−H) 2856, υ(C=O) 1747 υ(C−O) 1062; B10D90: υ(O−H) 3012, υ (arom. C−H) 2925, υ (alif. C−H) 2856, υ(C=O) 1747, υ(C−O) 1022; D100: υ(O−H) 3011, υ (arom. C−H) 2924, υ (alif. C−H) 2856, υ(C=O) 1747, υ(C−O) 1022.

### 3.3. Determination of inhibition time

Table 4 displays the results of the investigation carried out using the Rapid Small Scale Oxidation Test (RSSOT) as specified in the ASTM D7545 Standard Test Method for Oxidation Stability of Middle Distillate Fuels. Table 4 revealed a positive correlation between the content of antioxidants in biodiesel and its oxidation stability. The mean inhibition times were calculated as *y*mean = 47.83 min at 130 °C and *z*mean = 23.67 min at 145 °C, with corresponding standard deviations of s = 14.59 min and s = 4.52 min, respectively. These results confirm a pronounced reduction in oxidation stability with increasing temperature, while the inclusion of sulfenimide additives substantially extended the induction period relative to the neat biodiesel (D100) and base B10D90 blend.

### 3.4. Thermogravimetric analysis of sulfenimides

The thermal decomposition of compounds using thermogravimetric analysis techniques (TGA) was obtained during heating at 10 °C min^−1^ in a nitrogen atmosphere, and a summary of TGA results is illustrated in Table 5. All the compounds exhibited a single, sharp-step decomposition curve. Compound **1** was stable up to 202 °C. Then, the decomposition step started at 202 °C and ended at 351 °C. The maximum peak temperature was 322 °C, with an observed weight loss of 66.43% during the reaction. Compound **2** was thermally stable up to 201 °C and started decomposing between 201 and 325 °C. The maximum DTG peak temperature was 313 °C, with a weight loss of 94.93%. Compound **3** has the lowest thermal stability. For this sample, the decomposition temperature range was 153 °C to 259 °C with a maximum peak of 250 °C; the weight loss of the sample finished at nearly 100% at the temperature. Considering the decomposition processes’ onset and end temperatures, the order of thermal stability of compounds was **1** > **2** > **3**.

#### 3.4.1. Nonisothermal kinetics for sulfenimides

The nonisothermal kinetic analysis used the TG curves in a nitrogen atmosphere at four heating rates (5, 10, 15, and 20 °C min^−1^). To perform the kinetic study of sulfenimide thermal decomposition, the conversion rate α versus T values have been abstracted from TG curves obtained at different heating rates. The isoconversional kinetic method (model-free) assumes that conversion rates differ in each part of the overall conversion. That can be seen as the activation energy changes throughout the observed transformation [56]. It is observed that the TG curves tend to the right, and the reaction temperatures increase with the increasing heating rate of all samples. That results from variations in the decomposition temperature at higher heating rates [57]. Significant weight losses in all measurements were observed at room temperature (350 °C). Thermal decomposition takes place in the temperature range between 30 and 350 °C. Moreover, after this temperature, no reaction was observed in the TG curves for each experimental data point. Since this phase has also been identified in the sample and its decomposition occurs within the studied temperature range, it inevitably affects the kinetic curves [51].

**Figure 7:**
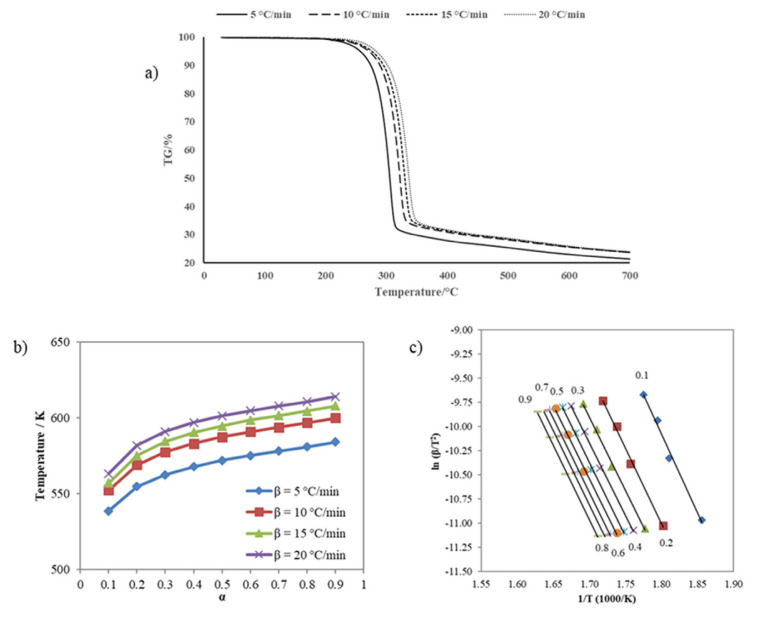
**(a)** TG curves of compound **1** at different heating rates, **(b)** Relationship between α and thermal decomposition temperature of compound **1** at various heating rates, and **(c)** Arrhenius plots for KAS method of compound **1**.

**Figure 8:**
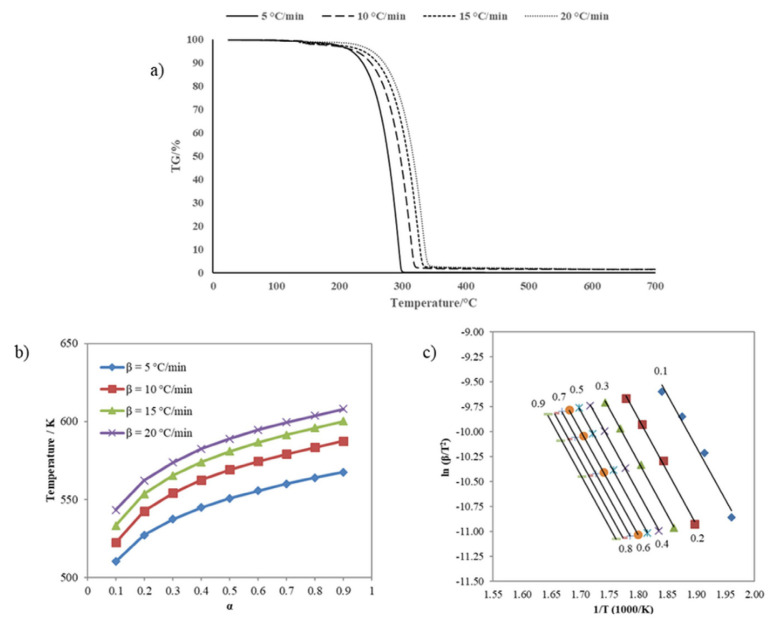
**(a)** TG curves of compound **2** at different heating rates, **(b)** Relationship between α and thermal decomposition temperature of the compound **2** at various heating rates, and **(c)** Arrhenius plots for KAS method of compound **2**.

Activation energies were calculated according to the KAS method, the model-free method used in the thermal decomposition of sulfenimides. Figure 7a depicts the TG curves at the four heating rates for compound **1**. Figure 7b shows the curves of temperature versus reaction rate. Figure 7c presents the diagrams plotted to calculate activation energy through the KAS method. According to the KAS method, the plots of ln β/T^2^ against 1000/T correspond to various conversions α calculated from the slopes of every line. The average activation energy value of compound **1** thermal decomposition calculated by the KAS method was 125.28 kJ mol^−1^ under nonisothermal conditions. Figure 8a presents the TG curves of compound **2** at various heating rates.

**Figure 9:**
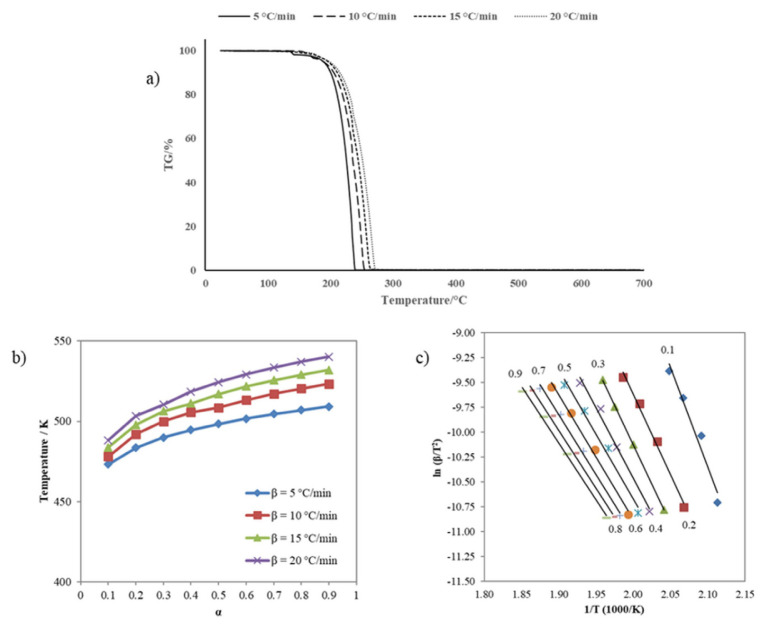
**(a)** TG curves of compound **3** at different heating rates, **(b)** Relationship between α and thermal decomposition temperature of compound **3** at various heating rates, and **(c)** Arrhenius plots for KAS method of compound **3**.

The kinetic parameters required to calculate the activation energy of compound **2** are shown in Figure 8b α-T data at different heating rates and Figure 8c Ln β/T^2^–1000/T graph for various α values. The average activation energy value of compound **2** decompositions obtained by the KAS method was 111.34 kJ mol^−1^. Figure 9a displays the TG curves of compound **3** at various heating rates. The relationship between α and T is illustrated in Figure 9b, and Arrhenius plots for the KAS method are given in Figure 9c. The average activation energy value of compound **3** decompositions calculated by the KAS method was 88.11 kJ mol^−1^.

The activation energy and thermal stability of compounds are directly proportional. The activation energy of a compound with high thermal stability is also high [58]. According to the activation energy values calculated above, the order is **1**>**2**>**3**. Thus, the activation energies obtained from the nonisothermal kinetic study and thermal analysis results confirm each other.

### 3.5. Evaluation of antioxidant activities

In living cell systems, antioxidants are critical chemicals that provide defense against reactive oxygen species (ROS). Reactive oxygen species (ROS) are reactive chemicals that radicalize and possess anionic charges, causing damage to DNA, RNA, and many biomolecular structures. These chemicals have the long-term potential to produce oxidative stress and diseases such as cardiovascular problems and cancer. Antioxidant molecules protect live tissue from these harmful reactive chemicals by inhibiting certain chemical reactions. Due to their strong antioxidant qualities, polyphenols such as phenolic acids, flavonoids, and ascorbic acid are critical for preserving good health. Antioxidant activity has drawn more attention lately [59]. Safe antioxidants are required to prevent food oxidative degradation and lower the risk of oxidative damage at the cellular level [60,61]. Nutrition can alter a living organism’s antioxidant defense mechanisms [62].

#### 3.5.1. Phosphomolybdenum activity

**Figure 10:**
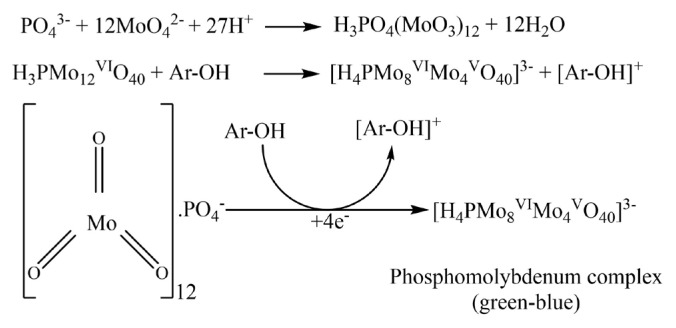
Reduction mechanism of ammonium molybdenum [53,63].

Phosphomolybdenum activity is based on the reduction of molybdenum from six to five, as seen in Figure 10 [53,63]. We investigated the reduction of molybdenum using different sulfenimide compounds. When the results in Table 6 were examined, the activity of the standard compound ascorbic acid used for comparison purposes was determined as 86.64 ± 0.66 μg/mL, while the sulfenimide containing methyl in the R group of sulfenimide compounds was determined as 83.59 ± 0.12 μg/mL. This result indicates that the activity was relatively close to that of the standard. Other sulfenimide compounds, in contrast, showed low activity.

#### 3.5.2. Reducing power capacity

Reducing power capacity is based on reducing iron from three to two, as shown in Equations 6 and 7. We investigated the reduction of iron using different sulfenimide compounds. When the results in Table 6 were examined, the activity of the standard compound ascorbic acid used for comparison purposes was determined as 45.42 ± 2.31 μg/mL, while the sulfenimide containing methyl in the R group of sulfenimide compounds was determined as 35.43 ± 3.09 μg/mL. This result demonstrates a higher activity compared to the used standard. Other sulfenimide compounds, in contrast, showed low activity. The reduction mechanism of Fe in the determination of reducing power is shown in Equations 6 and Equation 7 [55,64].


(6) $$2Fe{{\left(CN\right)}_{6}}^{3-}+C_{6}H_{8}{O_{6}}^{-}\to 2Fe{{\left(CN\right)}_{6}}^{4-}+C_{6}H_{7}O_{6}+H^{+}$$

(7) $$4Fe^{3+}\;3Fe{{\left(CN\right)}_{6}}^{4-}\to Fe_{4}{\left[Fe{\left(CN\right)}_{6}\right]}_{3}$$

#### 3.5.3. Mechanistic insight into the antioxidant behavior of sulfenimides

The antioxidant capacity of sulfenimide derivatives can be rationalized through their ability to interrupt radical chain reactions, a key mechanism underlying oxidative degradation in biodiesel systems. Structurally, sulfenimides are characterized by an N–S linkage, where the sulfur atom can serve as a radical acceptor or donor, depending on environmental oxidative conditions. The presence of the electron-deficient phthalimide ring adjacent to the sulfur atom further stabilizes radical intermediates via delocalization [11,21].

Proposed Mechanism [11,20,21]:

Initiation (Homolytic Cleavage): Under elevated temperatures or oxidative stress, the S–N bond in sulfenimide can undergo homolytic cleavage, producing a phthalimide-thiyl radical (Phth–S^•^) and a nitrogen-centered radical (R–N^•^). This is facilitated by the relatively weak S–N bond and the stabilization of the resulting sulfur-centered radical: The described reaction is given in Equation 8.


(8) $$Phth-S-NR\to Phth-S^{.}+N^{.}-R$$

Propagation (Radical Scavenging): The Phth–S^•^ species is a moderately stable thiyl radical that can effectively interact with peroxyl radicals (ROO^•^), commonly formed during lipid peroxidation of fatty acid methyl esters (FAMEs). This reaction leads to the formation of sulfenyl peroxides: The described reaction is given in Equation 9.


(9) $$Phth-S^{.}+ROO^{.}\to Phth-S-OOR$$

Which are thermally more stable and do not propagate further radical reactions.

Termination (Recombination): The remaining thiyl radicals can recombine to form disulfide bridges or other stable dimers, such as: The described reaction is given in Equation 10.


(10) $$2Phth-S^{.}\to Phth-S-S-Phth$$

Alternatively, radical recombination with carbon-centered or alkoxyl radicals can also occur, neutralizing propagating species and halting the chain reaction.

The phthalimide moiety plays a critical role in stabilizing sulfur-centered radicals by facilitating electron delocalization through its rigid, conjugated, and electron-withdrawing aromatic structure. This stabilization prolongs the lifetime of thiyl radicals (R–S^•^), thereby enhancing their reactivity toward neutralizing reactive oxygen species, particularly lipid-derived peroxyl radicals (ROO^•^) [11,21]. The radical-scavenging mechanism of sulfenimides closely parallels that of endogenous thiol-based antioxidants such as glutathione (GSH); however, the incorporation of the phthalimide core imparts superior thermal and oxidative stability. This is attributed to its planar geometry and resonance-assisted electron distribution, which reduce the susceptibility to premature degradation under elevated temperatures [11,20,21]. When incorporated into biodiesel–diesel blends, sulfenimide derivatives serve a dual function: (i) they effectively interrupt lipid peroxidation chain reactions, thereby mitigating oxidative degradation, and (ii) they modulate crystallization behavior at subzero temperatures by interfering with the molecular packing of fatty acid methyl esters. Collectively, these attributes contribute to a marked improvement in the oxidative and thermal resilience of biodiesel-based fuels during storage and operational conditions.

Table 7 presents the structure–activity relationship (SAR) of sulfenimides, offering a comparative overview of the substituent effects (electronic and steric) on their antioxidant activity. It supports the proposed mechanism by correlating molecular structure with phosphomolybdenum activity, reducing power capacity, and radical stabilization efficiency described above.

A rigorous comparison with Karakullukçu et al. (2024) situates the present sulfenimide results within the broader context of antioxidant research on biodiesel–diesel blends [65]. In that study, isatin–thiosemicarbazone (ISTSC) derivatives improved both the oxidative stability and cold-flow performance of biodiesel-diesel blends through radical trapping and thermal stabilization mechanisms, with Trolox serving as the standard antioxidant.

In our work, sulfenimide additives demonstrated a similar trend enhancing oxidation resistance and lowering crystallization temperatures despite differences in blend composition and kinetic modeling approaches. Both additive types operate through sulfur-centered radical stabilization, yet they differ structurally: ISTSCs are Schiff bases containing an azomethine (C=N) group, whereas sulfenimides possess an S–N linkage connected to a phthalimide ring. This variation influences radical delocalization and thermal behavior but preserves the overall antioxidant mechanism.

The observed parallel between the two systems and consistency with previously reported Schiff base and heteroatom based antioxidants confirm that sulfenimides act via analogous radical scavenging pathways while offering enhanced thermal robustness and structural simplicity for biodiesel stabilization.

## 4 Conclusions

This comprehensive study demonstrated that sulfenimide derivatives possess significant potential as multifunctional additives in biodiesel–diesel blends. The integration of sulfenimides not only enhanced oxidative resistance, as evidenced by prolonged inhibition times in RSSOT analysis, but also improved cold-flow behavior by reducing crystallization onset temperatures, as determined via differential scanning calorimetry (DSC). Among the tested compounds, CH_3_-substituted sulfenimide (**1**, CH_3_-Ph-S-Pht) exhibited superior antioxidant performance, attributed to its electron-donating and low steric hindrance properties that facilitate thiyl radical stabilization. In contrast, the Br-substituted derivative (**3**, Br-Ph-S-Pht) displayed the weakest antioxidant behavior, correlating with its electron-withdrawing and bulky substituent, which likely hinder radical interaction and delocalization.

Fourier Transform Infrared Spectroscopy (FT-IR) confirmed the successful incorporation of sulfenimides into biodiesel matrices through the observation of characteristic vibrational bands associated with both ester and antioxidant functionalities. Thermogravimetric analysis (TGA) and nonisothermal kinetic modeling using the Kissinger–Akahira–Sunose (KAS) method revealed that sulfenimides possess moderate to high thermal stability, with activation energies ranging from 88.11 to 125.28 kJ/mol. The order of thermal stability (**1** > **2** > **3**) matched both TGA decomposition profiles and kinetic energy barriers, providing a consistent structure–property relationship.

In vitro assays (phosphomolybdenum and reducing power capacity) further validated the antioxidant efficacy of sulfenimides, particularly the CH_3_-substituted compound (**1**), which demonstrated superior activity relative to the benchmark antioxidant, ascorbic acid. However, this comparison is intended solely to highlight the relative antioxidant response under identical experimental conditions and does not imply a broader industrial superiority beyond the tested reference. Mechanistic insights supported that sulfenimides function through a radical-trapping antioxidant mechanism, akin to that of thiol-based endogenous antioxidants such as glutathione, yet offer superior thermal stability due to the rigid phthalimide scaffold.

To the best of our knowledge, this is the first study to report the application of structurally defined sulfenimide derivatives as dual-purpose additives in biodiesel–diesel matrices, offering both oxidative and thermal improvements. Given their structural simplicity, synthetic accessibility, and multifunctionality, sulfenimides represent promising candidates for scalable and cost-effective biodiesel additive development. Future work should focus on long-term engine performance tests and the environmental impact assessment of sulfenimide residues to ensure safe and sustainable deployment.

## Figures and Tables

**Table 1:** The properties of ascorbic acid (AA) [24,26].

Property	Ascorbic acid (AA)	Chemical structure
**Molecular formula**	C_6_H_8_O_6_	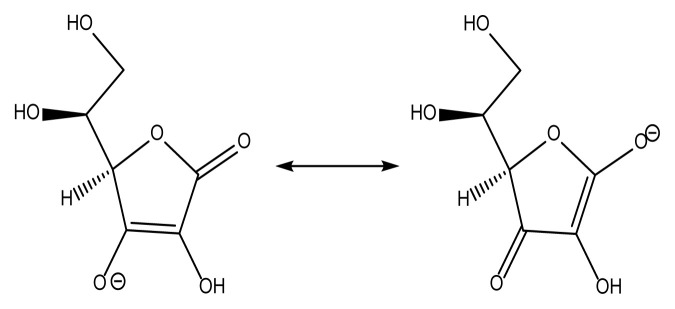
**Molecular mass (g/mol)**	176.124 g/mol	
**Density (g/cm^3^)**	1.65 g/cm^3^	
**Melting point (°C)**	190–192 °C	
**IUPAC name**	(5R)-[(1S)-1,2-Dihydroxyethyl]-3,4-dihydroxyfuran-2(5H)-one	
**Other names**	Vitamin C	
**Appearance**	White or light-yellow solid	

**Table 2:** Physical data of sulfenimide derivatives.

Compound code	R	Molecular formula	Molecular weight g/mol	Melting point (°C)	Yield%
**1**	4-CH_3_C_6_H_4_-	C_15_H_11_NO_2_S	269.32	202–204 (lit. 204–205) [37]	81
**2**	C_6_H_5_-	C_14_H_9_NO_2_S	255.29	158–161 (lit. 160–161) [21]	84
**3**	4-BrC_6_H_4_-	C_14_H_8_BrNO_2_S	334.19	185–187 (lit. 187–188) [38]	72

**Table 3:** D100, B10D90, B10D90_AA, B10D90_**3**, B10D90_**2**, and B10D90_**1** crystallization onset temperatures (°C) in a N_2_ atmosphere* and biodiesel-diesel sulfenimides mix amounts and codes.

Sample	Crystallizations onset temperature (*xi*) (°C)	(*xi* - *x*_mean_)^2^	Biodiesel (%)	Diesel (%)
D100	|−7.24|	0.51361	-	100
B10D90	|−7.64|	0.10028	10	90
B10D90_AA	|−7.89|	0.00444	10	90
B10D90_**3**	|−8.06|	0.01068	10	90
B10D90_**2**	|−8.39|	0.18778	10	90
B10D90_**1**	|−8.52|	0.31734	10	90

* Values are expressed as means (n = 2).

**Table 4:** The inhibition period of D100, B10D90, B10D90_AA, B10D90_**3**, B10D90_**2**, and B10D90_**1** can be influenced by the addition of sulfenimides and BHT*.

	ASTM D7545 Minutes (130 °C) (*xi*)	(*yi* − *y_mean_*)^2^	ASTM D7545 Minutes (145 °C) (y*i*)	(*zi* − *z_mean_*)^2^
D100	22 ± 0.01	667.94	11 ± 0.01	74.11
B10D90	42 ± 0.01	34.03	21 ± 0.01	7.11
B10D90_AA	50 ± 0.01	4.69	24 ± 0.01	0.11
B10D90_**3**	55 ± 0.01	51.89	26 ± 0.01	5.44
B10D90_**2**	58 ± 0.01	103.81	27.5 ± 0.01	14.70
B10D90_**1**	60 ± 0.01	148.28	28.5 ± 0.01	23.37

* Values are expressed as means (n = 2).

**Table 5:** Characteristic parameters of the thermogravimetric analysis of the sulfenimides.

Compounds	T_onset_/°C	T_max_/°C	Weight loss/%	Total Weight loss/%
**1**	202	322	66.43	76.85
**2**	201	313	94.93	98.12
**3**	153	250	99.58	100

T_max_ – Maximum peak temperature (DTG), Weight loss – Weight change during decomposition

**Table 6:** A_0.5_ value is the concentration at which an absorbance of 0.50 is effective for phosphomolybdenum activity and reducing power capacity.

Samples and standard	Phosphomolybdenum, A_0.5_ (μg/mL)	Reducing power, A_0.5_ (μg/mL)
CH_3_-Ph-S-Pht (**1**)	83.59 ± 0.12^a^	35.43 ± 3.09^a^
H-Ph-S-Pht (**2**)	150.84 ± 1.14^c^	88.86 ± 1.63^d^
Br-Ph-S-Pht (**3**)	223.84 ± 2.38^d^	73.52 ± 3.30^c^
Ascorbic acid	86.64 ± 0.66^b^	45.42 ± 2.31^b^

The letters a, b, c, and d are statistically significant indicators

**Table 7:** Structure–activity relationship (SAR) for sulfenimides.

Compound	Substituent (R)	Electronic -effect	Steric hindrance	Relative antioxidant efficiency
**1**	–CH_3_ (*p*-tolyl)	Weakly electron-donating (via hyperconjugation); stabilizes thiyl radicals moderately	Low (small methyl group, minimal ortho-hindrance)	High – Strong activity in both PMA (83.59 μg/mL) and RP (35.43 μg/mL)
**2**	–H (phenyl)	Electron-neutral; delocalization limited to the aromatic system	Moderate (planar phenyl ring without substituents)	Moderate – Average capacity (150.84 μg/mL in PMA; 88.86 μg/mL in RP)
**3**	–Br (*p*-bromophenyl)	Strongly electron-withdrawing (via inductive effect); destabilizes thiyl radicals	High (bulky and polarizable Br atom at para-position)	Low – Poor performance (223.84 μg/mL in PMA; 73.52 μg/mL in RP)
Ascorbic Acid	–	Strong electron donor; stabilizes radicals via resonance	Negligible (small, flexible molecule)	Benchmark – 86.64 μg/mL in PMA; 45.42 μg/mL in RP

## Data Availability

Data are contained within the article.
